# Measles outbreak investigation in Southwest Ethiopia, February 2017

**DOI:** 10.11604/pamj.supp.2018.30.1.15280

**Published:** 2018-05-18

**Authors:** Desta Hiko Gemeda, Hussein Mohammed Gena, Herbert Brian Kazoora, Haley McLeod

**Affiliations:** 1 Faculty of Public Health, Jimma University, Jimma, Ethiopia; 2 Haramaya University, Harar, Ethiopia; 3 African Field Epidemiology Network, Kampala, Uganda; 4 Rollins School of Public Health, Emory University, Atlanta

**Keywords:** Measles, outbreak investigation, Ethiopia

## Abstract

Effective response to complex disease outbreaks requires health workers to be equipped with the necessary knowledge and skills. This case study, based on an actual measles outbreak that occurred in Ethiopia in January 2017, was developed to enhance participants’ knowledge and skills on disease outbreak investigation and response using epidemiological study designs. This case study is prepared for health care workers with an advanced level of training in public health. This case study should be completed in a classroom setting and takes approximately 3 hours to complete.

## How to use this Study

**General instructions:** for this case study, two facilitators are needed for a class of 20 participants. It is recommended that participants work in teams. Facilitators should randomly select participants to read the narratives and questions. Divide participants into 4 or 5 groups to discuss questions. Then randomly select one participant from a group to summarize what they have discussed or come to the front of the classroom to write calculations on the whiteboard. When one group answers a question, move on to the next group to hear their input as well. When one person gives an answer, confirm it with participants in other groups. The facilitator should give a chance to all of the participants to participate, encourage dialogue between groups, and allow trainees to learn from each other.

**Audience:** national and sub-national surveillance officers, Field Epidemiology trainees and Master of Public Health graduates.

**Prerequisites:** for this case study, participants should have knowledge in the following areas: epidemiological study designs; Principles of outbreak investigation and response; Advanced data analysis; Sampling procedures in epidemiologic research.

**Materials needed:** laptop computer with Microsoft office, flip chart or white board.

**Level of training:** advanced level training in Public Health.

**Time required:** approximately 3 hours

**Language:** English

## Competing interest

The authors declare no competing interest.
